# Comorbidities, Depression Severity, and Circadian Rhythms Disturbances as Clinical Correlates of Duration of Untreated Illness in Affective Disorders

**DOI:** 10.3390/medicina57050459

**Published:** 2021-05-08

**Authors:** Giulia Menculini, Norma Verdolini, Francesca Brufani, Valentina Pierotti, Federica Cirimbilli, Agata Di Buò, Giulio Spollon, Filippo De Giorgi, Tiziana Sciarma, Alfonso Tortorella, Patrizia Moretti

**Affiliations:** 1 Department of Psychiatry, University of Perugia, 06132 Perugia, Italy; giuliamenculini@gmail.com (G.M.); f.brufani11@gmail.com (F.B.); valentina.pierotti@hotmail.com (V.P.); agata.dibuo@gmail.com (A.D.B.); giuliobreak87@hotmail.com (G.S.); tiziana.sciarma@unipg.it (T.S.); alfonso.tortorella@unipg.it (A.T.); 2 Bipolar and Depressive Disorders Unit, Hospital Clinic, University of Barcelona, IDIBAPS, CIBERSAM, Institute of Neuroscience, 08036 Barcelona, Spain; nverdolini@clinic.cat; 3 Section of Psychiatry, Clinical Psychology and Rehabilitation, Santa Maria Della Misericordia Hospital, 06132 Perugia, Italy; federica.cirimbilli@tiscali.it (F.C.); filippo.degiorgi@ospedale.perugia.it (F.D.G.)

**Keywords:** affective disorders, bipolar disorders, depressive disorders, affective spectrum, duration of untreated illness, comorbidities, depression severity, circadian rhythms

## Abstract

*Background and Objectives*: Affective disorders, namely bipolar (BDs) and depressive disorders (DDs) are characterized by high prevalence and functional impairment. From a dimensional point of view, BDs and DDs can be considered as psychopathological entities lying on a continuum. A delay in treatment initiation might increase the burden associated with affective disorders. The aim of this study is to analyze the correlates of a long duration of untreated illness (DUI) in these conditions. *Materials and Methods*: Subjects with BDs and DDs, both in- and outpatients, were recruited. Long DUI was defined according to previous research criteria as >2 years for BDs or >1 year for DDs. Socio-demographic, clinical and psychopathological characteristics of the recruited subjects were collected. Bivariate analyses were performed to compare subjects with a long and short DUI (*p* < 0.05). *Results*: In our sample (*n* = 61), 34.4% of subjects presented a long DUI. A long DUI was significantly associated with longer overall illness duration (*p* = 0.022) and a higher rate of psychiatric (*p* = 0.048) and physical comorbidities (*p* = 0.023). As for psychopathological features, depressive symptoms were more severe in the long DUI subgroup, as demonstrated by a higher score at the Clinical Global Impression-severity of depression (*p* = 0.012) item and at the anxiety/depression factor of the Positive and Negative Syndrome Scale (*p* = 0.041). Furthermore, subjects with a long DUI displayed more severe disruption of circadian rhythms, as evaluated by the Biological Rhythms Interview for Assessment in Neuropsychiatry total (*p* = 0.044) and social domain (*p* = 0.005) scores and by the Hamilton Depression Rating Scale diurnal variation items (18a: *p* = 0.029, 18b: *p* = 0.047). *Conclusions*: A long DUI may underpin higher clinical severity, as well as worse illness course and unfavorable prognosis in affective disorders. Intervention strategies targeting comorbidities, depressive symptoms and circadian rhythms may decrease disease burden in subjects with a long DUI.

## 1. Introduction

Affective disorders, namely bipolar disorders (BDs) and depressive disorders (DDs), are chronic and debilitating conditions with a high prevalence worldwide. Despite affective disorders have been traditionally categorized in BDs and DDs, a continuity between different affective disorders has also been supported according to the Kraepelinian model, describing “a common root with gradual transitions between the individual forms” [1]. The existence of an “affective spectrum” going from unipolar depression to bipolar mania [2] was also hypothesized on the basis of opposite polarity symptoms combining themselves in the context of mixed states [3,4], but also considering the high prevalence of lifetime subthreshold hypo/manic symptoms in subjects diagnosed with DDs [5] and the frequent misdiagnosis of subjects affected by bipolar depression as DDs [6]. The proposed dimensional model for affective disorders may present relevant clinical and therapeutic implications [7,8].

Timely intervention is considered as a predictor of better symptomatic and functional outcomes in both BDs and DDs [9]. Indeed, affective disorders occur during adolescence or young adulthood in almost of 60% cases [10,11]. Despite the possible presence of prodromes, recognizing such clinical features may be challenging, due to the retrospective nature of their evaluation and to possible differential diagnosis issues [12]. Subsequently, affective disorders and particularly BDs may remain undiagnosed for long periods of time [13]. Duration of untreated illness (DUI), defined as the time between the onset of affective symptoms and the first adequate pharmacological treatment, was investigated by a growing body of literature during the last decades [14,15]. This clinical characteristic appeared to be particularly relevant in BDs, which displayed a longer DUI when compared to DDs, schizophrenia, and anxiety disorders [16,17]. A long DUI in BDs, empirically defined as longer than 2 years [14,18], was associated with worse clinical and course characteristics, namely higher number of suicide attempts [14] and hospitalizations [13], more frequent relapses [19], and higher comorbidity rates [18]. Similarly, a DUI longer than 1 year showed a poorer outcome and course in DDs, with a higher rate of psychiatric comorbidities and more severe psychiatric symptoms during the following years [20]. A longer DUI may also affect recurrence rates in MDD [21], as well as response to antidepressant treatment [22,23].

The International Society for Bipolar Disorders (ISBD) enlisted DUI among possible useful clinical characteristics for the staging of BDs [24], further underlining the influence of diagnostic and treatment delay on illness course and prognosis. Despite this, given the heterogeneous nature of studies investigating DUI and the lack of univocal findings, this concept needs validation by additional research. Furthermore, possible factors contributing to diagnostic delay in such illnesses have not been fully clarified yet [25]. Moreover, to our best knowledge, literature confirming the impact of a lung DUI on functional outcomes in affective disorders is scanty, despite preliminary studies confirmed an association between a long DUI and general assessment of functioning (GAF) score and occupational status [13].

It should also be considered that several symptom domains may contribute to the clinical complexity of affective disorders, such as psychotic symptoms, mixed features, and impulsiveness [3,26,27]. During the last years, increasing attention has also been paid to circadian rhythm disturbances. Indeed, alterations in sleep-wake, eating, social, and activity patterns are frequent clinical features in the context of affective spectrum disorders [28]. Circadian rhythm disturbances were hypothesized to play a role in the pathophysiology of affective disorders, also underpinning alterations in the hypothalamic-pituitary-adrenal (HPA) axis [29]. In addition, disturbances in the above-mentioned systems were associated with a higher clinical severity, as well as with more prevalent subthreshold inter-episode symptomatology and worse treatment outcomes [30].

Given these premises, the aim of the present study is to analyze socio-demographic, clinical and psychopathological correlates of a long DUI in subjects with affective disorders. A better characterization of long DUI may help in identifying factors that could serve as future targets of treatment strategies in this population, also considering worse outcomes that may be underpinned by delayed treatment. Furthermore, the identification of potentially modifiable factors associated with a long DUI may help to reduce the gap between clinical onset, access to care and adequate management.

## 2. Materials and Methods

The present observational study was conducted between 1 April 2018 and 31 December 2020. Both inpatients and outpatients were considered for inclusion. Subjects were recruited at the Department of Psychiatry, Clinical Psychology and Rehabilitation of the General Hospital/University of Perugia, Italy, and at Community Mental Health Centers of the Local Health Units USL 1 and 2, Umbria, Italy. Subjects aged 18–65 with a diagnosis of BDs or DDs according to the latest edition of the Diagnostic and Statistical Manual of Mental Disorders (DSM-5) criteria were considered for inclusion. Exclusion criteria were the following: diagnosis of mental retardation, neurocognitive disorders, or physical illnesses possibly affecting mental status. Additionally, subjects without an adequate oral/written comprehension of Italian language were excluded.

Clinical assessments were conducted by psychiatrists with expertise in the field of affective disorders. Socio-demographic and clinical characteristics were collected by means of a specific form, with aid of electronic medical records. The latter also allowed us to confirm the presence of comorbid medical conditions, that were divided into the following groups: cardiovascular, endocrine, hematologic, infectious, neurologic, rheumatic, respiratory, and other diseases. According to previous literature on the topic, DUI was evaluated retrospectively by an operative criterion. Particularly, we calculated DUI by subtracting the age at onset to the age at first adequate pharmacological treatment, namely mood stabilizers or atypical antipsychotics for BDs and antidepressants for DDs [31,32]. We defined a long DUI as >2 years for BDs [13,14,18] and >1 year for DDs [33].

The Structured Clinical Interview for DSM 5- Clinical Version (SCID-5-CV) [34] was used in order to confirm the diagnosis of BDs or DDs and to evaluate psychiatric comorbidities. A trained clinician conducted the clinical interview with the subjects. For those who were diagnosed with BDs or DDs according to the structured interview, the remaining modules of the SCID-5-CV were administered, in order to assess the presence of the following comorbidities: anxiety disorders, obsessive-compulsive and related disorders, post-traumatic stress disorder, feeding and eating disorders, attention-deficit/hyperactivity disorder, and adjustment disorder. Personality disorders were evaluated by means of the Structured Clinical Interview for DSM-5 Personality Disorders (SCID-5-PD) [35]. Overall illness severity was assessed by means of the Clinical Global Impressions-Bipolar Disorder Version (CGI-BP), an instrument that maintains the original structure of the CGI focusing on specific components of BDs [36]. The severity of depressive, hypo/manic, and psychotic symptoms was evaluated by the Hamilton Rating Scale for Depression (HAM-D) [37,38], the Young Mania Rating Scale (YMRS) [39,40], and the Positive and Negative Syndrome Scale (PANSS) [41]. The PANSS has traditionally been divided into positive (items 1–7), negative (items 8–14) and general psychopathology (items 15–30) subscales. We applied a dimensional approach to the PANSS, considering a previous five-factor model including positive, negative, excitement, disorganized, and anxiety/depression factor [42]. Further psychopathological features, namely impulsiveness and circadian rhythm disturbances, were assessed by the Barratt Impulsiveness Scale-11 items [43] and the Biological Rhythm Interview for Assessment in Neuropsychiatry [44]. The BIS-11 is a self-report instrument composed of 30 items investigating different dimensions of impulsiveness that are described through three subscales: attentional (problems related to concentrating), motor (fast reactions, restlessness), and non-planning (orientation toward the present) impulsiveness. Higher scores at the BIS-11 suggest higher levels of impulsiveness [43,45]. The BRIAN is an interview consisting of 18 items measuring different circadian rhythms, namely sleep, social rhythm, overall activities, and eating behavior. All items are scored from 1 (no difficulties in maintaining circadian rhythms) to 4 (serious difficulties in maintaining the habitual rhythm), with greater total scores indicating more severe circadian rhythm disruption [44,46]. Finally, the Functioning Assessment Short Test [47] was administered in order to evaluate functioning of the included subjects in different areas. The FAST is a clinician-administered scale evaluating six different areas of functioning: autonomy, occupational functioning, cognitive functioning, financial management, social functioning, and leisure time. Scores can be determined for each of the considered areas, and items are scored from 0 (absence of difficulties) to 3 (high difficulty) [47,48].

All subjects signed informed consent for participating in the study, authorizing the use of personal data. The study was conducted according to the Declaration of Helsinki and followed good clinical practice guidelines. The research protocol was approved by the local ethic committee of the Umbria region (protocol number: 12958/18/ON, 22 March 2018). The collected information was recorded into an electronic database. Descriptive analyses were performed to evaluate the distributional properties of the analyzed variables in the sample. Subjects with a short and long DUI were compared by means of bivariate analyses (Mann–Whitney U or t-test for continuous variables depending on normality of distribution, chi-square test for categorical variables; *p* < 0.05). Normality was assessed by means of the Kolmogorov–Smirnov test. All analyses were performed with the Statistical Package for Social Science (SPSS) software, version 21.0.

## 3. Results

### 3.1. Sample Characteristics

During the considered period of time, 94 subjects were screened for inclusion in the study. Among these, one was excluded due to the presence of a physical illness (recent traumatic brain injury) that influenced the psychopathological status, while another one was diagnosed with mental retardation. Moreover, 31 subjects denied their consent to participate in the study. The study population consisted of 61 subjects, 51 (83.6%) inpatients and 10 (16.4%) outpatients, of which 44 (72.1%) were females. The mean age in the sample was 47.23 (±13.87) years old, ranging from a minimum of 19 to a maximum of 65. As for diagnostic features, 44 (72.1%) subjects were affected by BDs. A long DUI was detected in 21 (34.4%) subjects and the mean duration of DUI in the whole sample was 4.58 years.

### 3.2. Comparison of Socio-Demographic, Clinical and Course Carachteristics in Subjects with Long and Short DUI

Socio-demographic characteristics did not significantly differ between subjects with long and short DUI (see Table 1). Furthermore, no statistically significant differences were detected when comparing course characteristics among the two populations. Subjects with a long DUI presented a significantly longer illness duration (mean 23.30 ± 10.03 vs. 17.18 ± 14.66, *p* = 0.022). As for diagnostic features, subjects with a long DUI displayed a higher rate of psychiatric (66.7% vs. 40%, *p* = 0.048) and physical comorbidities (66.7% vs. 32.5%, *p* = 0.023), despite no significant differences were detected in the prevalence of single comorbidities. The most frequent psychiatric comorbidities were represented by substance-related disorders, borderline personality disorder, and anxiety disorders (33.3%, 23.8%, and 14.3% in the long DUI subgroup). As for medical comorbidities, the most frequent were endocrine diseases (35.7% among subjects with long DUI), followed by cardiovascular diseases (28.6%). The rates of DDs and BDs did not differ between the two subgroups and neither did the type of current affective episode.

### 3.3. Comparison of Psychopathological Features in Subjects with Long and Short DUI

Severity of depression as evaluated by CGI-BP was demonstrated to be higher in subjects with a long DUI (3.3 ± 1.46 vs. 2.29 ± 1.61, *p* = 0.012), who also showed a significantly higher mean score at the PANSS anxiety/depression factor (8.94 ± 2.84 vs. 7.25 ± 3.01, *p* = 0.041). Conversely, scores at the PANSS positive (8 ± 4.75 vs. 11.72 ± 5.96, *p* = 0.016) and disorganized factors (5.78 ± 2.51 vs. 8.08 ± 3.37, *p* = 0.014) were significantly lower in subjects with a long DUI. As for HAM-D score, a more significant variation in depression severity during the day was evaluated (HAM-D item 18a, 0.43 ± 0.5 vs. 0.20 ± 0.52 *p* = 0.047, and 18b, 0.62 ± 0.8 vs. 0.25 ± 0.64, *p* = 0.029).

No significant differences in impulsivity scores as assessed by BIS-11 were detected. Higher total score at the BRIAN was detected in the long DUI subgroup (48.94 ± 11.08 vs. 43.24 ± 10.53, *p* = 0.044), as well as a higher score in the BRIAN social domain (9.53 ± 2.57 vs. 7.32 ± 2.65, *p* = 0.005). Levels of functioning, as measured by the FAST, did not show significant differences between the two populations. Mean scores at the administered scales are displayed in Table 2.

## 4. Discussion

In this cross-sectional study, longer illness duration, higher rate of comorbidities, higher severity of depressive symptoms, as well as more severe circadian rhythms disruptions, demonstrated a correlation with a long DUI in BDs and DDs. On the contrary, positive symptoms and disorganization were inversely associated with a long DUI.

Affective disorders were evaluated as psychopathological entities lying on a continuum according to the “unitary” Kraepelinian approach [4,5], hypothesizing that DUI could present homogeneous correlates in unipolar and bipolar subjects. Interestingly, no differences in diagnostic characteristics between subjects with long and short DUI were detected, confirming that a longer DUI could represent a transdiagnostic clinical feature. Furthermore, the mean DUI did not significantly differ among BDs and DDs. This is not totally consistent with previous literature, showing longer treatment delay in BDs, possibly reaching 10 years or more [14,18]. Despite this, a progressive reduction of medium DUI for all serious psychiatric disorders was evidenced during the last years, possibly reflecting changes in social and cultural factors that may also influence the reduction of mental health-related stigma, with a subsequent higher tendency to seek help [15]. Moreover, greater attention was paid to early intervention in psychiatry throughout the last decade, which should also be considered in order to interpret our results [49].

No differences in actual age were detected in the present study, whilst illness duration was significantly higher in the long DUI subgroup, as demonstrated by previous research [50]. This could also suggest a higher number of mood episodes and thus represent an indirect index of higher clinical severity. Similarly, we evidenced a relevant association between DUI and comorbidities, which could also underpin a worse illness course [51]. This is consistent with previous literature that detected a longer DUI in subjects with BDs and comorbid anxiety [52] and personality disorders [18]. Given the high prevalence of such comorbidities [53] and poor functional outcomes that may be connected to them [54,55], the evaluation of psychiatric comorbidities should be mandatory when assessing subjects that report delayed treatment for affective disorders.

As for medical comorbidities, these are associated with longer illness duration in affective disorders, as well as with a reduction in overall functioning and quality of life [56]. Furthermore, physical comorbidities significantly contribute to the high mortality of subjects with serious mental illnesses [57]. A longer DUI may also influence lifestyle in this population [58], as demonstrated by the significant association between long DUI and tobacco smoke [59]. To note, physical comorbidities could affect response to pharmacological and psychotherapeutic interventions, acting as an indirect determinant of worse outcome in subjects that already received delayed treatment [60]. Interestingly, higher physical comorbidity rates and mortality in subjects with affective disorders may be linked to allostatic load processes [61]. Indeed, during affective relapses, the activation of physiological systems involved in the adaptation to internal and environmental factors, such as the immune-inflammatory system, may contribute to brain changes and to overall higher disability [62]. Conversely, pharmacological agents for the treatment of affective disorders exert a neuroprotective effect, confirming the influence of timely intervention on illness trajectories [12].

In the present study, a long DUI exerted an effect on the severity of depression. Similarly, in a previous study assessing predictors of depression severity in a longitudinal study [63] the strongest predictor of depression severity at follow-up, not depending on the duration of antidepressant treatment, was a long DUI. A possible explanation of this finding is the worse response to antidepressants that could result from a longer DUI [22,23]. This could be particularly interesting in consideration of the higher risk of developing cognitive impairment in depressed subjects with long DUI [64]. Furthermore, severity of depression was also demonstrated to represent an independent predictor of low quality of life in both BDs and DDs [65], suggesting the need for implementing early and adequate interventions focused on depressive symptoms in affective disorders. On the other side, subjects with a long DUI presented lower scores at the positive and disorganization factors of the PANSS, accounting for a lower severity of psychotic symptoms in this population. This is consistent with previous literature, demonstrating that psychotic and manic symptoms were associated with an earlier recognition of affective disorders and thus with a short DUI [18,66].

Circadian rhythm disturbances were also associated with a long DUI in the present sample, as demonstrated by higher BRIAN total score, higher score at the BRIAN social domain, and higher score at the HAM-D item 18 which evaluates “diurnal variations” of depressive symptoms. Particularly, the BRIAN social domain includes items assessing difficulties in communicating with others, as well as in adapting personal habits to those of cohabitants and in finding time to spend with family and friends, but it also evaluates the overuse of electronic devices. Circadian rhythm disruptions represent relevant clinical features of affective disorders and mood symptoms, also demonstrating a correlation with their underlying pathophysiology [28,67,68]. Such alterations were also identified as predictors of lower functioning in BDs, possibly mediating the disorder-related disability [69]. Furthermore, circadian rhythms and particularly social rhythms were demonstrated to be clinical correlates of metabolic syndrome in depressed subjects, leading to the hypothesis that biological rhythms and physical comorbidities may display a complex interaction in the context of affective disorders [70]. Under this perspective, DUI may represent a further element involved in this interaction, which should be clarified by future prospective studies.

In the present study, no significant associations were found with specific clinical characteristics, i.e., suicidality, repeated hospitalizations and functioning, which is not totally consistent with previous literature [13,33,71]. The adoption of heterogeneous criteria may determine disparities among study samples, thus partially explaining differences in our findings. Future studies based on the most recent treatment guidelines for affective disorders are expected to further clarify this issue.

The present study presents limitations. First, the small sample size and the cross-sectional design may limit the generalizability of findings. Moreover, during the Coronavirus disease 2019 (COVID-19) pandemic, only outpatient emergency visits were performed at the recruiting centers, which may have further affected the study sample. Not only may a larger study sample allow for a higher generalizability of findings in the future, but it could also permit the stratification of statistical analyses, i.e., presenting results for DDs and BDs separately. Second, the cross-sectional design did not allow us to infer causality and only permitted to find statistically significant associations among the considered independent variables and DUI. In addition, the retrospective evaluation of DUI, based on clinical information provided by in-/outpatients and their relatives, might have led to further biases. Indeed, although we had the possibility to validate clinical information by using electronic medical records, data concerning the onset of affective symptoms was not available in most cases since it referred to several years before the current evaluation. Finally, our sample was mainly composed of hospitalized patients, which could have contributed to a higher severity of the current clinical picture.

## 5. Conclusions

The present study confirms the early identification of affective disorders as a major issue, since a long DUI may underpin higher clinical severity in affective spectrum disorders, not depending on categorical diagnostic features. A significant association between long DUI and comorbidities could be responsible for a worse illness course and unfavorable outcomes. Furthermore, higher severity of depression and circadian rhythm disturbances underpin a lower quality of life in affective disorders. The timely diagnosis and treatment of both psychiatric and medical comorbidities should represent a priority in subjects with affective disorders with a long DUI. Additionally, treatment strategies aimed at reducing the severity of depressive symptoms and circadian rhythm disruptions might improve clinical outcomes in this population.

## Figures and Tables

**Table 1:** Socio-demographic characteristics of subjects with long (*n* = 21, 34.4%) and short (*n* = 40, 65.6%) DUI.

Socio-Demographic Characteristic (Yes Listed)	Long DUI (*n*, %)	Short DUI (*n*, %)	Χ^2^	*p*	Phi Coefficient
Female gender	15 (71.4)	29 (72.5)	0.000	1.000	−0.11
Italian nationality	20 (95.2)	37 (92.5)	0.000	1.000	0.053
Scholarity > 13 years	4 (19)	10 (25)	0.042	0.838	−0.067
Married/cohabitant	8 (38.1)	14 (35)	0.000	1.000	0.641
Single	13 (61.9)	26 (65)	0.000	1.000	−0.031
Employed	10 (47.6)	17 (42.5)	0.012	0.911	0.049
Living alone or with marital family	15 (71.4)	32 (80)	0.560	0.454	−0.097
	**Long DUI** (mean, SD)	**Short DUI** (mean, SD)	**Mann–Whitney U**	***p***	**η^2^**
Age	49.95 (12.18)	45.80 (14.62)	380.00	0.350	<0.001

Notes: DUI = Duration of untreated illness; SD = Standard deviation.

**Table 2:** Psychopathological characteristics of subjects with long (*n* = 21, 34.4%) and short (*n* = 40, 65.6%) DUI as measured by the administered scales.

Psychopathological Feature	Long DUI- Mean (SD)	Short DUI- Mean (SD)	Mann– Whitney U	t	*p*	η^2^	Hedges’ g
Affective symptoms							
YMRS total score	11.52 (10.47)	13.77 (11.44)	378.50	-	0.528	0.006	-
HAM-D total score	16.00 8.68)	12.10 (8.01)	-	−1.756	0.084	-	0.473
CGI-severity of depression	3.33 (1.46)	2.29 (1.61)	244.50	-	0.012	0.106	-
CGI-severity of mania	3.00 (1.89)	3.24 (1.75)	289.50	-	0.641	0.004	-
CGI total score	4.06 (0.75)	3.93 (1.02)	230.50	-	0.852	<0.001	-
**General psychopathology (PANSS)**							
Positive factor	8.00 (4.75)	11.72 (5.96)	195.50	-	0.016	0.096	-
Negative factor	10.67 (4.95)	10.64 (5.58)	301.50	-	0.670	0.003	-
Anxiety/depression factor	8.94 (2.84)	7.25 (3.01)	213.50	-	0.041	0.069	-
Excitement factor	8.00 (3.96)	9.86 (4.47)	240.00	-	0.121	0.040	-
Disorganized factor	5.78 (2.51)	8.08 (3.37)	192.50	-	0.014	0.100	-
Total score	56.22 (12.52)	63.40 (17.28)	-	1.729	0.091	-	0.454
**Impulsivity (BIS-11)**							
Attentional factor	16.84 (3.69)	17.03 (4.06)	-	0.166	0.869	-	0.048
Motor factor	22.58 (5.34)	21.59 (5.42)	-	0.783	0.536	-	0.183
Non-planning factor	26.42 (4.90)	26.90 (5.28)	-	0.895	0.755	-	0.093
Total score	65.84 (10.43)	65.17 (12.50)	-	−0.193	0.848	-	0.057
**Circadian rhythms (BRIAN)**							
Sleep domain	12.15 (4.02)	11.15 (3.56)	289.50	-	0.363	0.014	-
Activity domain	12.45 (4.60)	11.56 (3.89)	-	−0.760	0.451	-	0.215
Social domain	9.53 (2.57)	7.32 (2.65)	-	−2.935	0.005	-	0.843
Eating domain	7.94 (2.46)	8.38 (3.16)	-	0.511	0.612	-	0.149
Predominant activity domain	5.45 (1.96)	5.00 (1.76)	306.00	-	0.534	0.006	-
Total score	48.94 (11.08)	43.24 (10.53)	201.50	-	0.044	0.067	-
**Functioning (FAST)**							
Autonomy domain	5.58 (3.96)	4.55 (4.37)	305.00	-	0.338	0.015	-
Work functioning domain	6.58 (4.83)	7.47 (4.49)	-	0.692	0.492	-	0.191
Cognitive functioning domain	6.74 (4,56)	6.13 (4.98)	-	−0.455	0.658	-	0.126
Financial issues domain	2.63 (1.92)	2.45 (2.27)	342.50	-	0.749	0.002	-
Interpersonal relationships domain	9.53 (4.13)	7.84 (5.68)	295.00	-	0.262	0.021	-
Leisure time domain	6.26 (9.95)	7.21 (13.21)	322.00	-	0.503	0.007	-
Total score	37.32 (19.13)	35.66 (24.70)	-	−0.256	0.799	-	0.072

Notes: BIS-11 = Barratt Impulsiveness Scale—11 items; BRIAN = Biological Rhythms Interview for Assessment in Neuropsychiatry; CGI-BP = Clinical Global Impression—Bipolar Disorders version; DUI = Duration of Untreated Illness; FAST = Functioning Assessment Short Test; HAM-D = Hamilton Rating Scale for Depression; PANSS = Positive and Negative Syndrome Scale; SD = Standard deviation; YMRS = Young Mania Rating Scale.

## Data Availability

The data presented in this study are available on request from the corresponding author. The data are not publicly available due to privacy restrictions.
